# List of Diseases at the Bath City Dispensary, from October 1, to November 1, 1801

**Published:** 1801-12

**Authors:** 


					Diseases in the Bath City Dispensary, 4.QI
List of Diseases at the Bath City Dispensary, from O&ober r,
, to November i, 1S01.
Anafarca ------ i
Contufion - - - - - i
Cyft i
Cholera ------ 4.
Catarrh ------ 3
Chlorofis ------ 3
Diarrhoea ----- 2
Dyfenteria ----- 3
Eruptiones - - - - - 1
Er/fipelas - - - - - , - 1
Epilepfia ------ I
Febris (fimplex) - - - - 6
Fiftu^a in Ano - - - - I
Gallrodynia ----- 3
Hernia ------ 1
Xnt-ellinal Hemorrhage - - 1
Incurvated Spine - - - 2
Lepra - -- -- -- 1
Leucorrhcea ----- 1
Ophthalmia ----- 2
Pleuritis ------ 2
Pertuffis ------ 4
Phthifis ------ 7
Pulmonary Complaints - - z
Pfora - -- -- -- 2
Prolapfus Ani - - - - x
Rheumatifm Acute - - - 6
Synochus Biliofa - - - 41
Dyfenterica - 7
Pleuritica - - 3
Gaftritica - - 1
Scrofula ------ 1
Suppuration ----- 1
Scarlatina Anginofa - - - x
Swelled Leg ----- j
Tuffis - - - - t - . - 2
Ulcerated Legs - - - - 7
- In two cafes of the Synochus Biliofa Dyfenterica, the dis-
charge of blood from the inteftines was very confiderable j and
the patients were much reduced in confequence.. A deco&ion
of the Swietenia was given in both inftances, with obvious
advantages. A few grains of calomel and rhubarb were occa-
sionally adminiftered ; for although the evacuations are frequent
in thofe cafes, it often happens that there is an accumulation of
hardened fceces in the inteftines.

				

